# Relationship Between Total Body Adiposity Assessed by Dual-Energy X-ray Absorptiometry, Birth Weight and Metabolic Syndrome in Young Thai Adults

**DOI:** 10.4274/Jcrpe.1108

**Published:** 2013-12-12

**Authors:** Sirianong Namwongprom, Kittipan Rerkasem, Antika Wongthanee, Sakda Pruenglampoo, Ampica Mangklabruks

**Affiliations:** 1 Department of Radiology, Faculty of Medicine, Chiang Mai University, Chiang Mai, Thailand; 2 Department of Surgery, Faculty of Medicine, Chiang Mai University, Chiang Mai, Thailand; 3 The Research Institute for Health Sciences, Chiang Mai University, Chiang Mai, Thailand; 4 Department of Internal Medicine, Faculty of Medicine, Chiang Mai University, Chiang Mai, Thailand

**Keywords:** Body composition, fat mass, birth weight, metabolic syndrome, dual-energy x-ray absorptiometry

## Abstract

**Objective:** The aim of this study was to compare body fat distribution using dual-energy X-ray absorptiometry (DXA) in young adult subjects with metabolic syndrome (MS) with those without MS and also to determine whether a significant association existed between total body fat mass (FM) and MS along with the effect of birth weight.

**Methods:** This cross-sectional study was conducted on 393 young adult subjects (175 male, 218 female). Body mass index (BMI), waist circumference, blood pressure, triglyceride, high-density lipoprotein cholesterol and glucose levels were determined. Total body FM, lean mass (LM) and percentage of body fat (%BF) were assessed by DXA. Adult Treatment Panel III criteria were used for the diagnosis of MS.

**Results:** The prevalence of MS was 5.6% among this group of young adult subjects aged 18.5-21.8 years. Subjects with MS (n=22) had significantly higher values for weight, height, BMI, waist circumference, %BF, total body FM, total body LM, and regional FM and LM. There was no statistically significant difference in bone mineral density between the two groups. There was also no association between birth weight and MS. Multiple logistic regression analysis showed that every 5 kg of total body FM (OR 1.68; 95%CI 1.06-2.66) adjusted for gender, birth weight status, and total body LM were significantly associated with MS.

**Conclusion:** Total body FM measured by DXA was related to MS in Thai young adults. Thus, body composition analysis might have a role in the identification of subjects with MS status.

**Conflict of interest:**None declared.

## INTRODUCTION

Metabolic syndrome (MS) refers to a complex of multiple metabolic abnormalities associated with coronary artery disease (CAD) and diabetes (1,2,3,4) It is characterized by a constellation of metabolic disorders including abdominal obesity, dyslipidemia, increased blood pressure, and insulin resistance. Although the prevalence of obesity as defined by the World Health Organization (WHO) is relatively low in Asia compared to Western countries, MS is becoming a significant public health problem (5,6) A simple set of diagnostic criteria were proposed for the diagnosis of MS for routine clinical practice, including the identification of three or more of the following risk factors: increased waist circumference, increased serum triglyceride, reduced high-density lipoprotein cholesterol (HDL-C), elevated blood pressure, and elevated glucose. Not only the increased abdominal obesity, defined as increased waist circumference, but also the increased general obesity was associated with MS, cardiovascular disease, and type 2 diabetes (7). The association of obesity with cardiovascular risk and the insulin resistance syndrome is not only related to the degree of obesity, but also appears to be dependent on the body fat distribution. The assessment of general obesity can be obtained by body mass index (BMI) and other indices, such as fat mass (FM) and percentage body fat (%BF).

Body composition and fat content can be measured by hydrodensitometry, bioelectrical impedance, and dual-energy X-ray absorptiometry (DXA). Gold standard techniques to determine body composition, such as hydrostatic weighing and deuterium dilution, are costly and time-consuming. Therefore, alternative non-invasive methods of body composition assessment that are easier and safer to administer have been developed. DXA provides a reliable estimate of total body composition. This technique is quick, accurate, and carries a low risk of exposure to radiation (8,9,10,11). Several body composition indices are obtained by DXA such as FM, lean mass (LM), bone mineral density (BMD), %BF.

The purpose of this study was to determine if there was a significant association between total body FM and MS. To this end, body composition using DXA was evaluated and compared in young adult subjects with and without MS, individuals who were all the offspring of mothers who had participated in the Chiang Mai Low Birth Weight Study (CMLBWS) 20 years ago (12).

## METHODS

As part of the cohort study on the relationship between birth weight and MS using the previous data from CMLBWS, this study included 393 offspring from the previously published CMLBWS (12). The original objective of the CMLBWS, conducted in 1989-1982, was to investigate the prevalence and the risk factors of LBW in 2 184 pregnant Thai women. Of the original 2 184 CMLBWS maternal subjects, 770 were randomly selected to participate in this cohort study on the relationship between birth weight and MS and also for the assessment of body composition. For this selection, the women who had previously participated in the CMLBWS were tracked using a 13-digit identification number to identify their present status and also their current address. Invitation letters were sent to these mothers. The mothers who showed interest in the study and their offspring were also provided with information regarding the study. When both the mothers and the offspring understood and were willing to participate in the project, they were required to complete a consent form. Of the 770 subjects in the cohort group, only 418 of the offspring showed an interest, and 25 of these individuals were excluded due to the incompleteness of their biochemical data.

The study protocol was approved by the Ethics Committee of Faculty of Medicine, Chiang Mai University.

The height and weight of each subject were measured with subjects wearing a light robe and no shoes. The BMI was calculated as weight (kg) divided by height squared (m2). Waist circumference was measured from the narrowest point between the lower border of the rib cage and the iliac crest.

Blood pressure was measured twice from the right brachial artery in a sitting position following a five-minute rest period and using an automatic device. The average of these two measurements was used.

Blood samples (5 mL) for the measurement of blood glucose, triglyceride and HDL-C levels were collected from each subject following a 12-hour fast.

Body composition was measured by the DXA machine (Hologic Discovery A, Hologic Inc., Bedford, MA) equipped with software version 12.3. The machine was calibrated daily using a standard phantom provided by the manufacturer. Body composition parameters consisted of BMD, total body FM (kg), total body LM (kg), and %BF. The measurements were performed on total body, left arm, right arm, left leg, right leg, and trunk.

MS was defined according to a joint interim statement of the International Diabetes Federation Task Force in 2009 (13). Subjects were considered to have MS if they had three or more of the following abnormalities: abdominal obesity, elevated triglycerides (≥150 mg/dL), reduced HDL-C (<40 mg/dL in males and <50 mg/dL in females), elevated blood pressure [systolic blood pressure (SBP) ≥130 and /or diastolic blood pressure (DBP) ≥85 mmHg or previous treatment], and elevated fasting plasma glucose (≥100 mg/dL) or treated diabetes.

**Statistical Analysis**

All data were analyzed using the Stata® version 11.0 (StataCorp, Texas, USA). Continuous data are presented as means ± standard deviation (SD) or as numbers and percentages. All continuous variables in this study showed a normal distribution. Student’s t-test and Fisher’s exact test were used to test for differences and association as appropriate. Multivariate logistic regression was used to determine the association between selected parameters (including 5-kg stratum of total body FM, gender, birth weight status, and 5-kg stratum of total body LM and MS. Odds ratios (OR) and 95% confidence interval (CI) were presented for multiple regression models to examine the relationship between 5-kg stratum of total body FM and MS, adjusted for gender, birth weight status and total body LM. All statistical tests were two-tailed, and the statistical significance was defined as p-value of less than 0.05. 

## RESULTS

The study group comprised a total of 393 subjects. 55.5% of the study population were women. Mean and SD values for age, BMI and waist circumference in the total group were 20.4±0.4 years (range, 18.5-21.8), 21.1±3.9 kg/m2 (range, 14.2-34.3), and 75.8±10.3 cm (range, 50-115.5), respectively. The mean±SD for the MS components in the total group were: serum triglycerides 87.5±55.9 mg/dL; HDL-C 55.4±14.0

mg/dL; SBP 113.7±12.0 mmHg; DBP 72.7±10.8 mmHg; and fasting plasma glucose (FPG) 83.8±10.6 mg/dL. The prevalence of MS was 5.6% among these young adult subjects.

Basic characteristics and body composition parameters of the subjects with and without MS are presented in Tables 1 and 2.

Subjects with MS (n=22) had significantly higher weight, height, BMI, and waist circumference values. There was no statistically significant difference in age and gender between the two groups. Compared with subjects without MS, those with MS had significantly abnormal mean levels for all MS components (p<0.05 and <0.01), with the exception of FPG (p=0.19) (Table 1). The detailed differences in body composition parameters of the two groups are presented in Table 2. The mean total and regional body FM were significantly higher in those with MS compared to those without MS (p<0.001). Mean total and regional body LM values were also significantly higher in subjects with MS (p<0.05). There was no difference in BMD values between the two groups (p=0.21).

**Association of Total Body FM with MS**

We transformed total body FM and LM into 5-kg strata. Univariate logistic regression analyses were performed to identify the association between total body FM, total body LM, gender, and birth weight of the subjects (Table 3). The 5-kg increase in total body FM and LM were associated with an increased risk of MS (OR 2.18; 95% CI 1.65 to 2.89 and OR 1.51; 95% CI 1.22 to 1.87). The birth weight of the subjects was classified into 3 groups: normal 2.5 to 3.5 kg, low birth weight <2.5 kg, and high birth weight >3.5 kg. There was no association of MS with female or male gender and low or high birth weight (Tables 3 and 4). Multiple regression analyses were performed to examine the association of MS with total body FM (Table 4). After adjusting for gender, birth weight and total body LM, it was observed that a 5-kg increase in total body fat was significantly associated with increase in the risk of the MS (OR 1.68; 95% CI 1.06-2.66).

## DISCUSSION

To our knowledge, the present study is the first conducted in Thai young adult subjects that addresses the relationship between total body FM measured with DXA and MS. All subjects were young adults who were the offspring of mothers who had participated in the CMLBWS 20 years ago as a part of the cohort study on the relationship between birth weight and MS. These follow-up data in the young adult subjects were a valuable resource for exploring the relationship of MS with birth weight and total body FM. The estimation of MS in this study is based on specific reference data for Thai people. Therefore, the prevalence of MS in this study could be more accurate than using the Caucasian cut-off values. It is known for instance that waist circumference cut-off points are likely to be influenced by sex, ethnicity, and other factors (**14**). The prevalence of MS is age-dependent. In the present study, we observed a MS prevalence of 5.6% in 393 young adult offspring of the women who had participated in the previous CMLBWS (12). The result from the fourth National Health Examination Survey 2009 in Thailand showed that the prevalence of MS was 23.2% among adults aged ≥20 years and that the prevalence was higher in women than in men (26.8% and 28.5%). However, since these results were not age-specific, the real magnitude of the problem in the young adult group was not focused (15).

DXA is a new tool for assessment of body composition. It is a precise and easy method for total and regional body composition analysis. Many researchers studied the role of DXA in MS, however, most of the studies focused on the measurement of visceral fat rather than general obesity (16,17,18,19). The body composition measured by DXA consisted of three components: fat, lean, and bone. A significant difference was found in only two components, total body FM and LM, as shown in Table 2. No statistical difference of BMD between subjects with and without MS was demonstrated. Therefore, only total body LM was used for potentially confounding factor along with gender and birth weight.

Although the results of epidemiological studies suggested that the visceral obesity is a more important determinant of insulin resistance, diabetes and cardiovascular disease than generalized obesity (20), the results of the present study suggested that total body FM was associated with the risk of MS after adjusting for possible confounding factors. FM in all measured sites (total body, arm, leg, and trunk) in subjects with MS was higher than that in subjects without MS.

In univariate analysis, we found that both 5-kg total FM and 5-kg total LM had a relationship with MS (OR 2.18; 95% CI 1.65 to 2.89 and OR 1.51; 95% CI 1.22-1.87). However, once total body LM was taken into account with total body FM in multivariate analysis, the effect of total body LM was no longer present. A recent nested case-control study by Chien et al (21) found that body FM and percent body fat had a higher discriminative ability for the risk of MS than body LM. Final results after performing multivariate analysis indicated that 5-kg increments increased the total FM resulting in an increased risk of developing MS by 1.68 times. (OR 1.68; 95% CI 1.06-2.66). This might indicate that not only abdominal obesity, but also generalized obesity plays a role in MS and that the term general obesity should perhaps not be expressed in terms of BMI since LM is also a component of body weight.

An association between low birth weight and increased risk of MS in middle-aged adults and elderly has been reported in several clinical studies (22,23,24,25,26). However, the association in children and adolescents is still debatable. Controversial results have been reported on the association between birth weight status (low birth weight or high birth weight) and MS. Some studies demonstrated a strong relationship between low birth and high birth weight and the risk of MS (27,28,29,30,31,32), while other studies reported no association (33,34,35,36). In this present study, there was no association between either low birth weight or high birth weight and MS.

Several limitations of this study must be noted. First, the study subjects were not truly randomly sampled from the cohort. Secondly, this was a cross-sectional analysis, and thus, we have limited ability to establish the time related relationship between total body FM and MS. On the other hand, we believe that the appropriate analysis of the data has provided us with useful information in identifying the relationship between total body FM and MS.

In conclusion, total body FM was found to be related to MS in Thai young adults. Thus, body composition analysis may prove to be useful in the identification of subjects with MS. Longitudinal studies will be helpful in better characterizing this relationship and its implications for public health planning and management.

## ACKNOWLEDGEMENTS

We are grateful for the wiling cooperation of all participants. We also would like to thank Dr. Pien Chiowanich and his co-investigators in the 1990 study. This work was supported by joint funding from the Thailand Research Fund and the Commission of Higher Education (MRG 5280229). This research was also funded by the Faculty of Medicine, Chiang Mai University, Chiang Mai, Thailand. 

## Figures and Tables

**Table 1:**
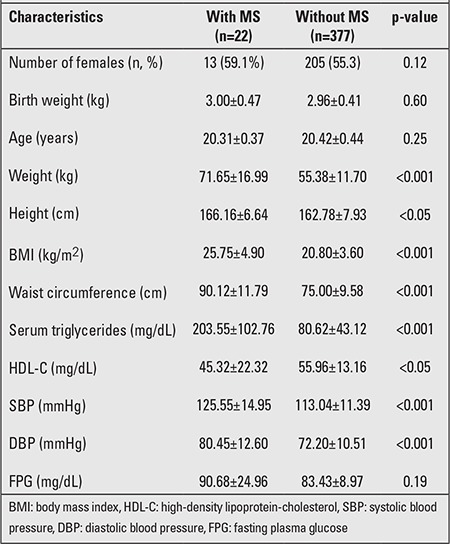
Baseline characteristics in 393 subjects with and withoutmetabolic syndrome (MS)

**Table 2:**
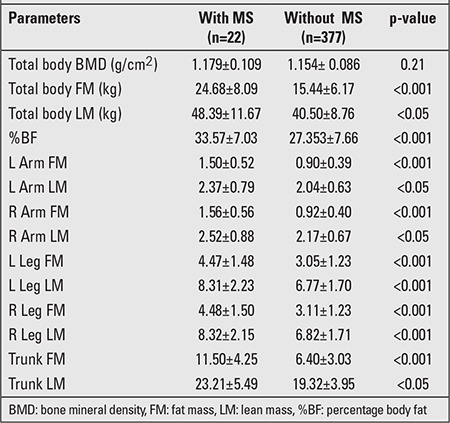
Comparison of body composition parameters in subjects with and without metabolic syndrome (MS) (mean ± SD)

**Table 3:**
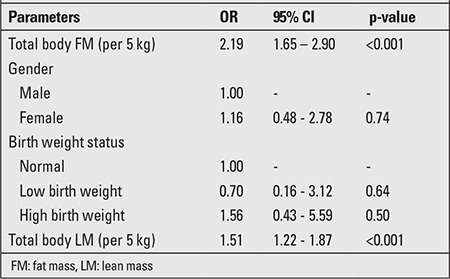
Univariate logistic regression analysis of the association between selected parameters and metabolic syndrome

**Table 4:**
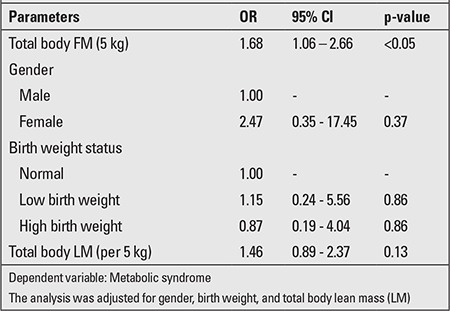
Multiple logistic regression analysis of the association between per 5 kg of total body fat mass (FM) and metabolic syndrome
